# Serological Evaluation for Measles among Italian and Foreign Medical Students in a University Hospital in Rome

**DOI:** 10.3390/vaccines11071256

**Published:** 2023-07-19

**Authors:** Luca Coppeta, Cristiana Ferrari, Giuseppina Somma, Viola Giovinazzo, Ersilia Buonomo, Marco Trabucco Aurilio, Michele Treglia, Andrea Magrini

**Affiliations:** 1 Department of Biomedicine and Prevention, University of Rome Tor Vergata, 00133 Rome, Italy; luca.coppeta@ptvonline.it (L.C.); giuseppina.somma@ptvonline.it (G.S.); viola.giovinazzo@ptvonline.it (V.G.); ersilia.buonomo@uniroma2.it (E.B.); andrea.magrini@uniroma2.it (A.M.); 2 Faculty of Medicine, University “Our Lady of Good Counsel”, 1000 Tirana, Albania; 3 Department of Medicine and Health Sciences “V. Tiberio”, University of Molise, 86100 Campobasso, Italy; marco.trabuccoaurilio@unimol.it; 4 Forensics Department, University of Rome Tor Vergata, 00133 Rome, Italy; michele.treglia@uniroma2.it

**Keywords:** occupational epidemiology, infection, health care workers, measles, foreign medical students

## Abstract

Background: Measles infection in the hospital setting is a major issue. Despite the availability of an effective vaccine, measles outbreaks continue to occur in some European countries. We aimed to evaluate the immunological status of medical students attending the Tor Vergata Polyclinic (PTV). Methods: Measles antibodies titers were assessed by venipuncture on a sample of 2717 medical students who underwent annual health surveillance visits from January 2021 to March 2023. Subjects showing serum IgG values above 1.0 S/CO were considered serologically protected. Personal data, country of origin, and main demographic characteristic were also collected. Results: 66.7% (1467 Italian and 346 foreign) of medical students showed protective IgG antibodies levels. Female students were serologically immune more frequently than males (68.6% vs. 63.3%; *p* < 0.01 at Chi2). The mean antibody titer was 1.72 S/CO, significantly higher in females than males (1.67 vs. 1.75, respectively; *p* < 0.05), and significantly related to age (*p* < 0.01). Albanian students, who were the largest foreign population in our study, showed a low serological protection rate (40/90: 44.4%). Conclusions: The proportion of serologically non-immune students is high, raising concerns about the possible risk of hospital transmission. Substantial differences in the rate of immunity have been found between subjects coming from different parts of Europe and the world. Pre-training assessment of all medical students and vaccination of susceptible individuals is highly recommended, particularly for those from low immunization rate countries.

## 1. Introduction

Measles is a highly contagious infectious diseases caused by an enveloped, RNA virus of the genus *Morbillivirus* (Paramyxoviridae family). At least 20 different genotypes have been isolated worldwide, although measles is serologically monotypic. Measles is highly contagious, and an infected person can transmit the virus to over 90% of unprotected close contacts [1].

After entering the airways via contaminated droplets, measles virus replicates in the upper respiratory tract of infected individuals and spreads through the blood to the other tissues (skin, eye, and respiratory tract) 5–7 days after exposure. Following an incubation period, infected patients experience symptoms (fever, cold, and conjunctivitis). After another 3–4 days, subjects develop a typical rash spreading from the face to the trunk and extremities. Patients with measles infection may spread the infection during the incubation period when the typical manifestations may be absent. In recent decades, measles has become less common, so it can be challenging for physicians to diagnose the infection even in cases of rush onset [1].

Most patients recover from measles without lasting effects, but severe forms with complications (e.g., otitis, pneumonia) may occur both in children and in adults.

Transmission of measles in the hospital setting is a major threat due to the possible spread of the infection to the most susceptible patients. Health care workers (HCWs) are considered to be at increased risk of measles infection due to their occupational exposures. According to the ECDC, hospital operators are 13 times more at risk than the general population [1] and represented a significant proportion of cases during recent measles outbreaks involving HCWs [2,3,4,5]. Nosocomial transmission of measles has also been reported in recent studies from Italian hospitals [6,7,8].

Prompt identification and isolation of measles cases, contact screening, and post exposure prophylaxis are the recommended measures to prevent large hospital outbreaks, but they cannot completely prevent nosocomial transmission due to the presence of asymptomatic patients and the route of indirect airborne transmission. Therefore, immunization of susceptible operators represents a crucial measure to prevent the circulation of the virus, both in operators and in the entire population. The measles vaccine is 97% effective in preventing measles when two doses of Measles Mumps and Rubella (MMR) are administered 12 months apart, while one dose is 93% effective. The World Health Organization (WHO) has promoted an immunization plan aimed at eliminating measles and rubella infections worldwide [9,10,11,12]. Since the basic reproductive number (R0) for measles is 12–18, a percentage of immune subjects of 92–94% is needed to reach herd immunity in the general population. However, given the risk of occupational exposure, the ideal HCW immunity rate is 100% in order to avoid nosocomial spread of the virus. According to the Advisory Committee on Immunization Practices (ACIP), all HCWs should have presumptive evidence of measles immunity (documented two doses MMR vaccination) or serological evidence of protective antibodies. In Italy, the vaccination coverage rate for MMR decreased compared to 2012, and in 2017, vaccination coverage among children fell below 85%, resulting in a large national outbreak with over 5000 infections occurring. The Italian government promoted a vaccination law that introduced compulsory vaccination for all school-age children. Although HCWs accounted for a large proportion of the 2017 measles cases [13], vaccination was not made mandatory for those operators, and immunization was only recommended in the Italian National Plan for Immunization and Prevention. Furthermore, despite a clear epidemiological shift towards older children and young adults in the distribution of cases by age, no active immunization campaigns targeting this age group have been undertaken.

In the European Region, the annual number of confirmed measles cases has been in the thousands since 2001, and in 2017 the EU experienced a resurgence of measles with 28 countries involved, several outbreaks, and 37 reported deaths, most of the cases occurring in Romania (5608), Italy (5098), Greece (967), and Germany (929). Measles cases continued to be reported across the EU region during the years 2018 and 2019 (17,822 and 13,200 cases, respectively). During 2020, measles cases decreased in all EU countries (6252 cases) due to social distancing, lockdown, and the other preventive measures taken for COVID-19, but in 2021, cases increased again, and over 21,000 were reported [14].

Globally, there were 159,067 measles cases and 60,700 deaths in 2020, as reported by WHO. Notably, 61 million doses of measles-containing vaccine have been delayed or missed due to delays related to immunization activities related to the COVID-19 pandemic. This fact has raised concerns about a possible future increase in the number of cases and deaths.

According to the European Center for Disease Control and Prevention (ECDC), most measles outbreaks occurring in Europe are the result of measles imported from another European or non-EU country [14].

Italy, as well as 33 other European countries, participates fully in the Erasmus program, the EU exchange and mobility program which supports university students from the EU and partner countries from all over the world to study in Europe. Medical students from countries with high measles circulation rates starting their medical training may represent a possible measles transmission vehicle for their colleagues and hospital patients.

The main aim of this study is to serologically evaluate the measles susceptibility rate among foreign medical students attending their internship at a university hospital in Italy.

## 2. Methods

To assess the serological status of students starting their internship at the Tor Vergata Policlinic in Rome, we collected the serological and demographic data of all Italian and foreign medical students who carried out a pre-training assessment at the occupational medicine service in the study period (from 1 January 2022 to 1 March 2023).

The list of medical students was taken from the certificates transmitted to the health directorate during the study period.

All study participants underwent venipuncture to the cephalic vein, median cubital vein, or basilic vein to draw blood for routine blood tests. A 10 mL blood sample was collected and delivered to the laboratory to detect measles-specific IgG antibodies. A semi-quantitative assessment of specific IgG antibodies was obtained by the Alifax VIRCLIA^®^ KIT VIR VCM054 Measles IgG assay, which uses chemiluminescent immunoassay (CLIA) technology with sensitivity and specificity values of 98% and 100%, respectively. Serum IgG values were expressed as signal-to-cut-off ratio (S/CO); values above 1.0 S/CO were considered protective based on actual evidence.

The records relating to the period from 1 January 2022 to 1 March 2023 were extracted from the laboratory software (Modulab^®^, version 3.1.02).

All measles-specific IgG antibody values were collected in a Microsoft Excel worksheet. Both Italian and foreign medical students were evaluated.

The personal data collected in the software consisted of name, age, and gender. The country of origin, not registered directly by the software, was obtained from the tax code of each subject; for subjects coming from the EU region (which represents the majority of reports), we identified three areas: Northern, Southern, and Eastern Europe. Italian students were classified separately, as they constituted the largest group. Vaccine data were not available for most of the study subjects, and therefore we did not collect them.

All participants underwent a physical examination and history, and subjects with acute symptoms of any type of disease were excluded from the venous blood test and deferred to a later time. We also excluded from the study individuals with incomplete serological data or those who tested positive for measles-specific IgM antibodies.

We then compared the mean values of the specific IgG antibodies of the protected students divided by age group through multivariate analysis.

### Statistical Analysis

The characteristics of the subjects were reported in numbers and, as a percentage, by types of variables. The difference in percentages between groups was assessed with the Χ2 test or Fisher’s Exact Test when appropriate. T-tests and ANOVA were used to evaluate differences between continuous variables. Pearson’s correlation tests were used to measure the statistical relationship, or association, between continuous variables.

A *p* value < 0.05 was considered statistically significant. All analyses were performed in SPSS version 25.0 for Windows.

The Ethics Committee for Research in Human Subjects of the Tor Vergata Policlinic hospital approved the study (approval no. 133/21).

## 3. Results

We evaluated the medical records of 2717 medical students (963 males and 1754 females). The mean age was 23.9 years (range: 18–27); 2164 subjects came from Italy, while 553 came from 47 other countries (see Table 1). All continents of the world, except Australia, were represented in the study population. The majority of foreign students came from European countries (365), while Asian (141), North American (16), and South American (16) students were less numerous, and only 16 subjects came from Africa.

Among European foreign students, 231 were from eastern Europe (Albania, Romania, Moldova, Hungary, Poland, Ukraine, Czech Republic), 105 from the Mediterranean Europe (Spain, France, Greece, Cyprus), and 58 from Northern Europe (Germany, Switzerland, Holland, Finland, Sweden, Norway). The origin of the population is shown in Table 2.

We found that 1813 students (66.7%) had a protective IgG antibodies level against measles. Female students were serologically immune more frequently than their male colleagues (68.6% vs. 63.3%; *p* < 0.01 at Chi2). The main results are reported in Table 1 and Table 3.

The mean antibody titer was 1.72 S/CO and was significantly higher in women than in men (1.67 vs. 1.75, respectively; *p* < 0.05 via ANOVA test). The measles antibodies titers were significantly related to the age of the operators (Pearson’s < 0.01).

Next, we assessed the rate of serologically immune students by area and state of origin. Regarding the geographical area, subjects from North America (US and Canada) showed the highest rate of protective antibodies, while the lowest rate was found in Asian operators. In particular, half of these last students (76 operators) came from Iran.

In the EU region the serological immunity rate ranged from 25% of Moldovan students (only eight subjects were present in the database) to 100% in many countries (France, Switzerland, Portugal, Malta, Russian Federation, Lithuania). The serological immunity rate among Italian students was 67.8%. Furthermore, Albanian students were the largest population among foreign students (90 subjects), and 50 of them (55.5%) showed a non-protective antibody titer.

Overall, a similar rate of serologically immune workers was found among the tree EU regions: Mediterranean, North, East (*p* = n.s. at χ^2^ test).

Data are shown in Table 2.

## 4. Discussion

The main aim of our study was to evaluate the rate of serologically immune subjects in a large population of medical students, both Italian and foreign.

We found a high proportion of serologically non-immune individuals among those young operators, raising concerns about the need for a systematic immunization campaign for those trainees. Both the protected and mean antibody titer rates were significantly higher in female students than in male students. It is well known that the immune response to vaccines and natural infection is gender-dysmorphic, and furthermore in many countries the prevention of congenital rubella syndrome may have resulted in a higher proportion of girls receiving the MMR vaccine than their male peers.

Most of the study subjects were Italian, but individuals from all regions of the world were included in the analysis. Some countries were represented by only a few operators, and continents other than Europe (and partly Asia) had fewer than 20 operators each, making the data of little significance. Keeping this limitation in mind, some considerations can be drawn from the analysis.

The rate of serologically unprotected individuals was similar across European regions, even though some countries represented 100% immune students, probably due to different local immunization policies. Recent reviews of EU immunization policies for HCWs have shown significant differences between countries in terms of recommended programs, application frame (mandatory or recommendation), target groups, and health-care context [15]. These heterogeneous policies significantly affect the immunization rate in all countries. In previously published studies, suboptimal immunization rates for some important vaccine-preventable diseases have been reported among European HCWs, including those operators employed in high-risk settings [16,17,18,19].

Despite the relatively small number of enrolled subjects, the data from Eastern Europe are, in our opinion, indicative, especially as regards Albanian operators. These students are the most represented foreign population as a result of a collaboration agreement between the Policlinic of Rome and the University of “Nostra Signora del Buon Consiglio” of Tirana. Albanian students showed a lower serological protection rate than their Italian colleagues. Many Eastern European countries have similar organizational patterns in immunization programs, often based on compulsory childhood vaccination [20]. In Albania, although a large number of vaccinations are mandatory in childhood and are to be carried out in health facilities, recent studies have revealed a strong concern about the safety and efficacy of vaccines [21]. A large measles outbreak occurred in 2018–2019, with almost 1700 reported cases highlighting that is difficult to maintain optimal immunization coverage in that country [22,23]. The recent SARS-CoV-2 pandemic has caused a decline in the childhood vaccination rate [24,25] as well as a long persistent disturbance in the adult and occupational vaccination service, whose efforts have mainly been directed towards COVID-19 vaccination, affecting the other routine prevention activities, including immunization for vaccine-preventable diseases [26].

WHO recommends that 95% is the rate needed to prevent the spread of measles among susceptible individuals, and ACIP has recommended mandatory immunization policies for healthcare operators, including medical students [27]. However, in the present study, the subjects’ immune rate was less than 70%, raising concerns regarding the risk of nosocomial measles infection for those operators [28,29,30]. Indeed, because of their job tasks, HCWs have a higher risk of measles exposure and infection than the general population, and medical students may be exposed in the same occupational setting as other older workers.

In Italy, there is no mandatory national measles vaccination policy for medical students and health professionals, except in two Italian regions, and non-immune people are sometimes offered vaccination in the workplace but are more often directed to a public service immunization program, resulting in a delay or failure to administer the vaccine. In a previous study, direct vaccine administration was shown to be both more effective and cost saving than other strategies [31].

Given the retrospective nature of the study, it was not possible to evaluate a previous natural measles infection or MMR vaccination among serologically unprotected subjects; therefore, the real susceptibility to measles of the whole population is not clear. However, we can hypothesize that the rate of serologically unprotected students is similar to that of those susceptible to measles. In fact, in most European countries, the rate of naturally immunized subjects can be considered negligible given the epidemiological data on the circulation of measles after 2000. Regarding the vaccination rate, childhood immunization for measles was introduced in different periods in Europe, but the vaccination coverage of the population showed significant differences between countries, being generally higher in Northern Europe than in the Mediterranean area and in Eastern Europe. More than 90% of vaccinated subjects have been shown to have detectable levels of measles antibodies for measles 17 years after vaccination [32], and it is unlikely that a decline in immunity has occurred in those young subjects.

In light of the results of our study, to prevent a resurgence of measles in the European region, it is urgent in all countries to identify persistent immunity gaps and missed doses of MMR vaccine due to the COVID-19 pandemic, which may have left many groups in the population susceptible to this disease. Sustaining at least 95% routine coverage will stop transmission of the virus, so raising awareness among stakeholders and healthcare professionals about the risk of the disease is crucial.

In Italy, the measles vaccination coverage rate was less than 50% before 2000, and a law on compulsory vaccination for school-age students was enacted in 2017 [33]. We can therefore hypothesize that subjects born after 2000 (aged < 22 years) must have both a presumed immunity and detectable serological protection since, for reasons explained above, it is unlikely that the antibody titers have vanished in five years for those subjects vaccinated at school age. In previous studies we found a high level of serological immunity among medical students vaccinated with two doses of MMR more than 15 years after vaccination [34]. In previously published studies, a high serological susceptibility rate and a paradoxically higher risk of infection were found among young adults compared to other age groups [30,35,36]. Our data raise questions about the need for mandatory vaccination among Italian HCWs, especially those aged 25–30.

Given the inadequate vaccination coverage in Italy and the circulation of the measles virus [2,3], we recommend vaccination in childhood according to the vaccination schedule, serological evaluation, and possible vaccination for HCWs who do not declare this type of vaccination or do not have protective antibody titers [37]. This strategy has been found in previous studies to be highly cost effective and cost saving [31,35,38].

This paper has many possible limitations.

First, the study focused on serological evaluations, and we considered operators showing an IgG titer ≤ 1.0 S/CO as unprotected. It has been reported that following a complete response to vaccination, the mean level of measles antibodies naturally tends to wane over time, but immunological memory continues to provide long lasting protection, even in the absence of a detectable antibody titer. We also had no previous vaccination records. Therefore, the rate of unprotected students may have been overestimated because a proportion of serologically non-immune subjects may still be protected from measles infection, having developed a complete primary immunological response to the vaccine. However, according to recent findings, the rate of waning immunity after a two-doses MMR vaccination has been shown to decrease slowly over time, so we can assume that the young age of the study population makes complete loss of antibody protection unlikely [39].

Second, the circumstances of vaccination (type of vaccine administered, vaccination schedule, storage conditions, etc.) might have varied between study countries, resulting in different vaccine immunogenicity.

Third, the epidemiology of measles in the various regions of the world is heterogeneous, and the effect of a natural booster can consequently differ, depending on the likelihood of coming into contact with the virus.

Fourthly, students involved in international exchange programs may not be fully representative of the population of origin, as they probably belong to the upper social class, or they may have received vaccination in preparation for the period of study abroad. Finally, the different exposure risk according to the various hospital departments was not considered.

On the other hand, the strengths of our study are represented by the large sample size of medical students included in the study population, the comprehensive evaluation of measles immunity in different regions of the world, and the serological evaluation, which allowed for complete and accurate measles immunity in those young operators.

## 5. Conclusions

The results of our study highlight a worrying proportion of medical students who are serologically non-immune to measles at the start of their hospital internship. The measles immunity rate was heterogeneous in relation to the country of origin, raising concerns about the high risk of measles transmission among students from areas with the lowest immunization rates.

Pre-training assessment of all medical students regardless of their nationality and vaccination of unprotected individuals should be promoted in hospital infection prevention policies. Vaccination refusals and non-responder operators should be excluded from high risk settings.

## Figures and Tables

**Table 1:** Main population characteristics.

Characteristics	N (%)	Mean Age ± SD	*p* Value
Students	2717 (100)	23.9	
**Gender**			
Male	963 (35.4)	23.95 ± 2.23	n.s.
Female	1754 (64.6)	23.85 ± 2.23	
**Nationality**			
Italian	2164 (79.6)	24.02 ± 2.23	<0.01
Foreign	553 (20.4)	23.36 ± 2.16	
**Anti-Measles IgG titer**			
>1.0 S/CO	1813 (66.7)	23.68 ± 2.2	<0.01
≤1.0 S/CO	904 (33.3)	24.29 ± 2.2	

**Table 2:** Proportion of subjects with anti-measles IgG titer > 1.0 S/CO by country of origin.

Countries		Students with IgG > 1.0		
	Total Number	N of Protected	% of Protected	95% C.I.
Albania	90	40	44.4	34.0–55.3
Brazil	8	2	25	3.2–65.1
Bulgaria	6	2	33.3	4.3–77.7
Cameroon	8	4	50	15.7–84.3
Czech Republic	14	10	71.4	41.9–91.6
France	10	10	100	69.1–100
Germany	31	16	51.6	33.1–69.8
Greece	40	32	80	64.4–90.9
Iran	76	30	39.5	28.4–51.4
Israel	8	4	50	15.7–84.3
Italy	2164	1466	67.8	65.7–69.7
Moldova	8	2	25	3.2–65.1
Poland	24	20	83.3	62.6–95.3
Portugal	6	6	100	54.1–100
Romania	42	30	71.4	55.4–84.3
Russia	6	6	100	54.1–100
Slovakia	14	12	85.7	57.2–98.2
Spain	28	24	85.7	67.3–96.0
USA	14	14	100	76.8–100
Switzerland	6	6	100	54.1–100
Turkey	30	8	26.7	12.3–45.9
Ukraine	9	6	66.7	29.9–92.5
Hungary	16	16	87.5	79.4–100
Others	60	49	81.66	69.6–90.5
**Total**	2717	1813	66.7	64.9–68.5

**Table 3:** Immunological protection in relation to main study variables.

Variables		Students with IgG > 1.0		*p* Value
	Total Number	N of Protected	% of Protected	
**Gender**				
Male	963	610	63.3	<0.01
Female	1754	1203	68.6	
**Age**				
>24	1216	724	59.6	<0.01
≤24	1501	1088	72.4	
**Nationality**				
Italian	2164	1467	67.8	n.s.
Foreign	553	346	62.6	

## Data Availability

Data is unavailable due to privacy.
